# Real-time Classification of Non-Weight Bearing Lower-Limb Movements Using EMG to Facilitate Phantom Motor Execution: Engineering and Case Study Application on Phantom Limb Pain

**DOI:** 10.3389/fneur.2017.00470

**Published:** 2017-09-11

**Authors:** Eva Lendaro, Enzo Mastinu, Bo Håkansson, Max Ortiz-Catalan

**Affiliations:** ^1^Biomechatronics and Neurorehabilitation Laboratory, Department of Electrical Engineering, Chalmers University of Technology, Gothenburg, Sweden; ^2^Integrum AB, Mölndal, Sweden

**Keywords:** phantom limb pain, virtual reality, myoelectric control, electromyography, pattern recognition, neurorehabilitation, phantom motor execution

## Abstract

Phantom motor execution (PME), facilitated by myoelectric pattern recognition (MPR) and virtual reality (VR), is positioned to be a viable option to treat phantom limb pain (PLP). A recent clinical trial using PME on upper-limb amputees with chronic intractable PLP yielded promising results. However, further work in the area of signal acquisition is needed if such technology is to be used on subjects with lower-limb amputation. We propose two alternative electrode configurations to conventional, bipolar, targeted recordings for acquiring surface electromyography. We evaluated their performance in a real-time MPR task for non-weight-bearing, lower-limb movements. We found that monopolar recordings using a circumferential electrode of conductive fabric, performed similarly to classical bipolar recordings, but were easier to use in a clinical setting. In addition, we present the first case study of a lower-limb amputee with chronic, intractable PLP treated with PME. The patient’s Pain Rating Index dropped by 22 points (from 32 to 10, 68%) after 23 PME sessions. These results represent a methodological advancement and a positive proof-of-concept of PME in lower limbs. Further work remains to be conducted for a high-evidence level clinical validation of PME as a treatment of PLP in lower-limb amputees.

## Introduction

Following an amputation, it is common for the patient to perceive the missing limb as if it is still part of the body. The phenomenon, known as phantom limb, is accompanied by a wide range of sensory perceptions that can vary among patients but are collectively referred to as phantom sensations (such as warmth, cold, or kinesthesia) (1). Amputees can often experience painful sensations in their phantom limb, giving rise to a condition commonly known as phantom limb pain (PLP). The pathogenesis of PLP is still controversial, and there is currently no treatment regarded as generally effective. Therefore, PLP remains a major clinical challenge (2, 3).

Recently, promising results on the treatment of PLP were achieved with a novel technology tested on subjects with upper-limb amputation (4). This treatment, firstly introduced by Ortiz-Catalan et al. in 2014 (5, 6), aims at promoting the execution of phantom movements, and hence the name phantom motor execution (PME). Other contemporary research efforts have brought about a number of non-pharmaceutical initiatives to treat PLP focusing on voluntary or imagined phantom movements (7–11). PME distances itself form these approaches by the certainty it provides of phantom movements being actually executed, while visualized as direct biofeedback with unperceivable delay. This is achieved using a myoelectric pattern recognition (MPR) system that renders virtual and augmented reality (VR/AR) environments under the control of the subject’s phantom limb. For instance, a virtual arm superimposed on a live video projection of the patient’s stump can be controlled in a similar way as the patient’s arm prior to amputation. The advantage of such a system is twofold. First, the ease of movement of the virtual limb is a direct consequence of naturalistic muscular patterns of activation owing to the nature of MPR. Second, VR and AR environments provide visual feedback that is congruent with the phantom motion executed, thus facilitating motor execution (12, 13). Clinically significant improvements on PLP (approximately 50% reduction) found in upper-limb amputees treated with PME (4) call for this technology to be explored in lower-limb amputees suffering the same condition.

For many decades, MPR has been vastly studied for upper limbs (14), while advances for lower limbs are relatively recent and mostly focused on improving prosthetic control under weight-bearing conditions (15–20). However, in the context of implementing *PME* for lower limbs, the interest in MPR lies in non-weight-bearing conditions because the patient should be able to execute leg movements while sitting in front of a screen. More importantly, such movements must be natural, not the result of reaction forces. MPR for the non-weight-bearing condition has been attempted in offline (21) and real-time studies (22). Notably, Hargrove et al. demonstrated the discrimination of eight leg movements (knee flexion/extension, ankle plantarflexion/dorsiflexion, hip rotation medial/lateral, and tibial rotation medial/lateral) in both non-amputee and amputee subjects by recording surface electromyography (sEMG) signals with bipolar electrodes placed over nine residual thigh muscles (22). The adopted procedure for electrode placement and signal collection can be challenging in a rehabilitative setting. Primarily, not all muscles might be available depending on the level of amputation. Furthermore, anatomical changes following amputation could make it difficult to precisely identify the desired muscles.

**Figure 1:**
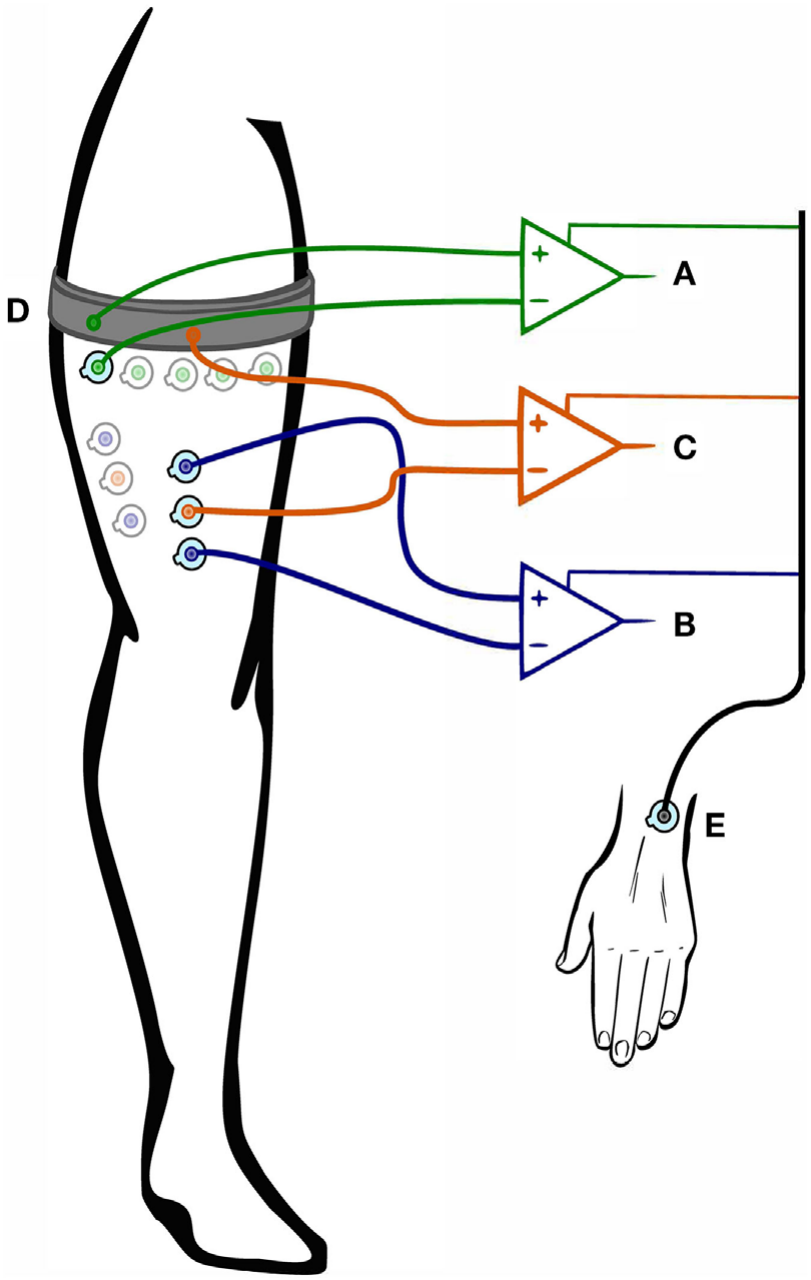
Sketch of the three electrode configurations. **(A)** Untargeted monopolar configuration, **(B)** targeted bipolar configuration, **(C)** targeted monopolar configuration, **(D)** common circumferential electrode, and **(E)** reference electrode.

We previously proposed two electrode configurations to acquire sEMG for MPR of non-weight-bearing movements of the lower limb (Figures 1A,C) (23). We compared these electrode configurations with the conventional bipolar targeted configuration in terms of signal-to-noise ratio (SNR) and offline MPR classification accuracy. We found that equally spacing the electrodes round the most proximal third of the thigh is a viable alternative to bipolar recordings from specific muscles, with the additional advantage of facilitating the recording procedure. However, MPR offline accuracy does not necessarily correspond with real-time performance (24–26). In this work, we validated previous offline findings using real-time metrics and performed the first clinical evaluation of *PME* on a lower-limb amputee who suffered from chronic, intractable PLP.

Ethical approval for the studies was granted by the ethical committee of Västra Götalandsregionen. The participants in both studies signed informed consent statements. The patient who underwent *PME* treatment was also informed of possible increases in pain, and uncertainty of positive outcomes.

## Materials and Methods

### Part I: Classification of Non-Weight-Bearing Lower-Limb Movements

#### The Subjects

Twelve non-amputees (five males and seven females, ages 23–30) and two amputees participated in the study. One amputee had a unilateral transfemoral amputation (70 years old and 35 years after amputation), whereas the other had a unilateral, transtibial amputation (72 years old and 22 years after amputation). The transfemoral amputee was trained in using the MPR system, while the transtibial amputee was a novice.

#### Electrode Placement

Non-amputees sat on a raised seat, allowing their feet to hang freely. This precaution was taken to ensure that patterns used for discriminating movements of the foot (ankle plantarflexion/dorsiflexion) were not generated by ground reaction forces. In one experimental session, sEMG signals using a targeted bipolar configuration (TBC) and a targeted monopolar configuration (TMC) were simultaneously acquired. In a different session, an untargeted monopolar configuration (UMC) was used (Figure 1). Amputees participated in both experimental sessions on two different days, and non-amputees were randomly divided into the two sessions (six each). Figure 1 shows the recording configurations as follows:

- *UMC* (Figure 1A): a circumferential electrode made of conductive fabric (silver-plated knitted fabric) was dampened with a small amount of water to decrease skin-electrode impedance and tied around the most proximal third of the thigh. Sixteen Ag/AgCl adhesive electrodes (disposable, pre-gelled Ag/AgCl, 1-cm diameter) were placed below the band (more distally on the leg) and equally spaced around the thigh. The gap between the electrodes and the band was approximately 4 cm. Differential measurements were recorded between each of the electrodes and the common circumferential electrode (CCE) (Figure 1D). The configuration is monopolar, due to the use of the CCE as a reference for the other adhesive electrodes.
- *TBC* (Figure 1B): eight pairs of pre-gelled electrodes were placed over the following eight muscles at an inter-electrode distance of 4 cm: sartorius, tensor fasciae latae, vastus medialis, rectus femoris, vastus lateralis, gracilis, the long head of the biceps femoris, and semitendinosus. The stump of the transfemoral subject was long enough to identify all the muscles.
- *TMC* (Figure 1C): for each pair of electrodes in the TBC, a third electrode was placed in between. The CCE was dampened and tied around the proximal third of the thigh. We recorded differentially between each of the eight electrodes and the average potential of the area covered by the CCE.

A reference electrode used for all recording configurations was placed on the contralateral wrist over the distal end of the ulna (Figure 1E).

#### Recording Session

The system used for sEMG acquisition was developed in-house and based on the RHA2216 chip (Intan Technologies, USA), with embedded filter (a third-order, Butterworth, low-pass filter with cutoff at 750 Hz and a first-order, high-pass filter with cutoff at 1 Hz). The system amplified the myoelectric signals from 16 channels with a gain of 200 times, and digitalized them with 16 bits of resolution at a 2-kHz sampling rate. Before proceeding to data acquisition, sEMG signals from all channels were checked to ensure the devise was functioning correctly. The data acquisition, signal treatment, pattern recognition, and real-time evaluation all used an open-source software (BioPatRec) for decoding motor volition using MPR (25).

**Figure 2:**
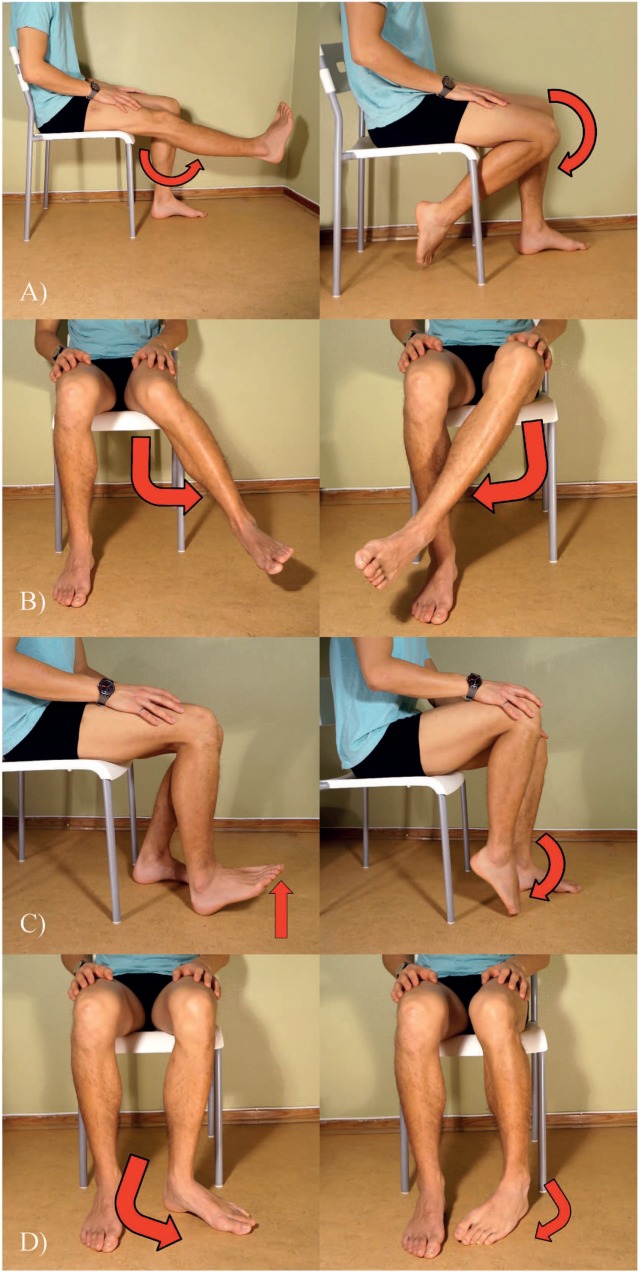
Photographs depicting the trained motions **(A)** knee extension and flexion, **(B)** femoral rotation outwards and inwards, **(C)** ankle plantar flexion and dorsiflexion, and **(D)** and tibial rotation outwards and inwards.

The participants were instructed to follow a graphical user interface showing the movements to be performed (Figure 2), along with a progress bar signaling the duration of each contraction. The recorded movements were as follows: knee flexion/extension, ankle plantarflexion/dorsiflexion, hip rotation medial/lateral, and tibial rotation medial/lateral. The amputees were asked to execute the movements as naturally as possible, focusing on their phantom leg. All participants were also instructed to perform the movements at a comfortable speed, avoiding abrupt contractions or jerks, as these would introduce motion artifacts in the signals. Once participants reached the end of their range of motion, they held the position for the remaining part of the contraction time, and then relaxed. For each movement, sEMG signals were collected in three consecutive repetitions of 4 s each, in which each repetition was followed by 4 s of rest. The subjects were asked to execute the movements at approximately 70% of their maximal voluntary contraction (according to their subjective estimation) to prevent premature fatigue. Before proceeding with the actual data collection, each subject executed one preparatory recording session to become familiar with the system. The recordings are available online in the repository of bioelectric signals of BioPatRec, under the name *8mov16chLowerLimb* (27).

#### Signal Treatment

Data recorded during the contraction time usually contain absent or transient sEMG signals due to a delay between the movement prompt and the actual execution, or anticipatory relaxation of the muscles. We reduced the impact of ambiguous information by discarding 15% of the signal at the beginning and at the end of the contraction time. This yielded trimmed contraction periods of 2.8 s each, which were then concatenated resulting in 8.4 s of total contraction signal. The signal obtained was subsequently divided, or segmented, into time windows of 200 ms, with 50 ms time increment. The segmentation produced 163 time windows for each movement, and from each time window four sEMG signal features were extracted per channel (mean absolute value, wave length, slope changes, and zero crossings) (28). The features extracted from all channels in a given time window formed a feature vector. The 163 features vectors corresponding to each time window were then randomly assigned to the classifiers’ training, validation, and testing sets in the following respective proportions: 40, 20, and 40% (25).

#### Classifier Training and Real-time Evaluation

The “rest” condition was considered as a movement or class, resulting in a classification task of nine patterns. Linear Discriminant Analysis in a One-Vs-One topology (LDA-OVO) was used for classification (5, 6). Immediately after the classifier was trained, the real-time performance in each electrode configuration was evaluated with the Motion Test (29), as it is implemented in BioPatRec (25). The Motion Test asks subjects to execute the trained movements that are presented to the user in random order Subjects performed the test twice. The following metrics were then evaluated:

- *Selection time*: time elapsed between the first prediction different from rest and the first correct prediction. The shortest selection time possible was 211 ms (200 ms of the first time window plus the processing time before the prediction is available).
- *Completion time*: time elapsed between the first prediction different from rest (as in the selection time) and the 20th correct prediction. The shortest completion time possible was 1.16 s.
- *Completion percentage*: the percentage of motions that were completed; or the motions that reached 20 correct predictions before the 10 s timeout.
- *Real-time accuracy*: only calculated for completed motions and accounts for the number of predictions needed to obtain 20 correct predictions. For example, if the completion time took 25 time windows, the real-time accuracy would be 80%.

The order in which Motion Tests were performed was randomized within the TBC and TMC groups. Two conditions were evaluated in random order with the UMC session: all 16 channels; and a subset of equally spaced 8 channels.

#### Statistical Analysis

We investigated the real-time performance of two alternative electrode configurations (TMC and UMC) to the conventional, TBC. Testing for statistical significance was conducted only on the non-amputees owing to the small sample size of the amputee group, in which case-only descriptive statistics were used. The TBC and TMC configurations were investigated on the same subjects, and the classifier for the real-time classification task was trained using data collected within the same recording session. Consequently, the two groups were compared by using the Wilcoxon signed-rank test. The UMC configuration was analyzed on a different set of subjects. The comparison between TBC and UMC with 8 channels (UMC-8 ch), and the one between UMC-8 ch and TMC were performed with Wilcoxon rank sum test for independent samples. In addition, UMC was investigated in two variants, with 8 and 16 channels, to determine if additional channels could improve performance, as tested with Wilcoxon signed-rank. Statistical significance was considered at *p* < 0.05 with Bonferroni correction.

### Part II: Case Study on a PLP Sufferer

#### The Subject

**Figure 3:**
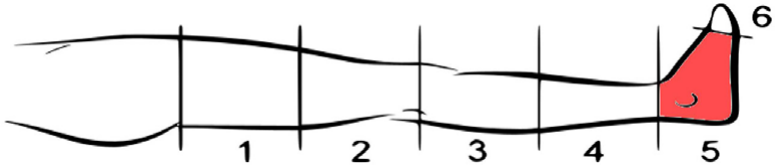
Representation of the phantom limb pain location in the lower-limb amputee subject treated with *phantom motor execution*.

A 70-year-old male with traumatic transfemoral amputation (unilateral) took part in the pain treatment case study. The subject described his phantom leg as of the same length as his normal leg and located the phantom pain in the foot (Figure 3, location 5). The PLP had been present since the amputation 35 years ago. However, the overall pain intensity had increased over the years, despite the implantation of a spinal cord neurostimulator 10 years prior to the start of our investigation. The participant described the pain as sustained low intensity pain, mainly present during the day, and recurrent high intensity pain, predominant in the evenings and at night. During periods of strong pain, the subject would feel the need to stand up, walk around, and use the neurostimulator. As a result, his sleep was disturbed by pain seizures that would wake him up and make him unable to sleep for more than 2 h per night.

#### The *PME* Treatment

The patient received *PME* interventions twice per week, for a total of 23 sessions. Each session lasted approximately 2 h, starting with pain assessment and continuing with *PME*. PLP was also monitored at 1, 3, and 6 months after the last treatment session.

**Figure 4:**
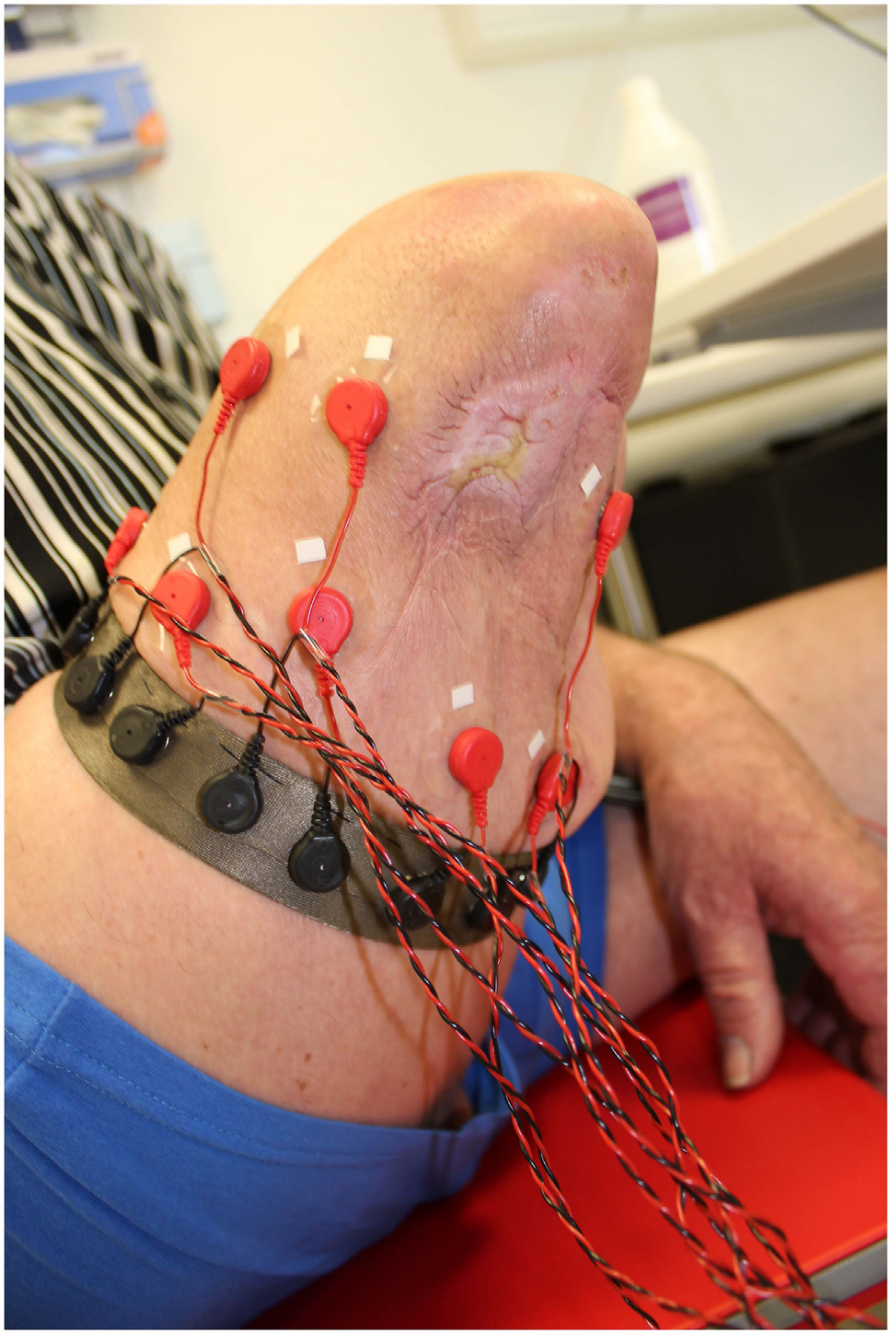
Example of targeted monopolar configuration used for the *phantom motor execution* treatment of the patient with lower-limb amputation.

After the pain interview, electrodes were placed on the stump. Initially the treatment was conducted with 16 electrodes in the TMC configuration (see Part I: Classification of Non-Weight-Bearing Lower-Limb Movements). However, after few treatment sessions, the muscles of the stump increased in size, producing stronger signals. Consequently, the electrodes were gradually reduced to eight (the subject preserved his ability to control the virtual environments). The location of the electrodes was determined by palpation while requesting the patient to move his phantom leg. Figure 4 shows an example of the TMC configuration used.

Different phantom movements (or set of movements) were exercised at an increasing level of difficulty as done in the upper limbs [see appendix of Ortiz-Catalan et al. (4) for details]. Myoelectric signals associated with the chosen set of movements were recorded to train the MPR system with LDA-OVO topology. The patient then practiced *PME* in virtual reality (VR), to later perform target achievement control (TAC) tests (30). The TAC test consists of executing the trained motions to control a virtual limb to match random target postures presented on the screen. The target postures reflected the previously trained 1 degree-of-freedom movements, as well as combinations of these to achieve multiple degrees of limb motions. The level of difficulty of the exercise depended on the number of movements trained, the type of movement, and if these were executed simultaneously. For example, distal movements are generally harder to control. On the other hand, consistent with our working hypothesis that *PME* reverts the central and peripheral maladaptive changes that took place following amputation, we aimed at exercising movements of the part of the phantom limb perceived as painful, which is commonly distal, as in the case of this patient.

#### Pain Assessment

The pain assessment interview was conducted at the beginning of each session and at 1, 3, and 6 months after the end of the treatment. We assessed changes in intensity, quality, and duration of PLP with a questionnaire derived from the Swedish version of the Short Form of the McGill Pain Questionnaire (SF-MPQ) (31) and study-specific questions. Specifically, the Numeric Rating Scale from 0 (no pain) to 10 (worst possible pain) was used to evaluate the intensity of pain at the moment of the interview. Moreover, quality and intensity of pain was assessed by the Pain Rating Index (PRI), as per SF-MPQ (32), and was calculated as the sum of the individual scores given to the pain descriptors. Furthermore, the time-varying pain profile of an average day was captured by a study-specific metric, the weighted pain distribution (WPD) (4–6), which required the patient to estimate the percentage of the time awake spent at each level of a 6-point scale (none to maximum, 0–5). The results of the questionnaire were then summarized in the WPD, which is the weighted sum of the pain scores. PLP location and length of the phantom limb were also monitored at each session. Finally, the patient was free to self-report comments regarding any aspect of the treatment, pain perception and quality of life.

## Results

### Part I

**Figure 5:**
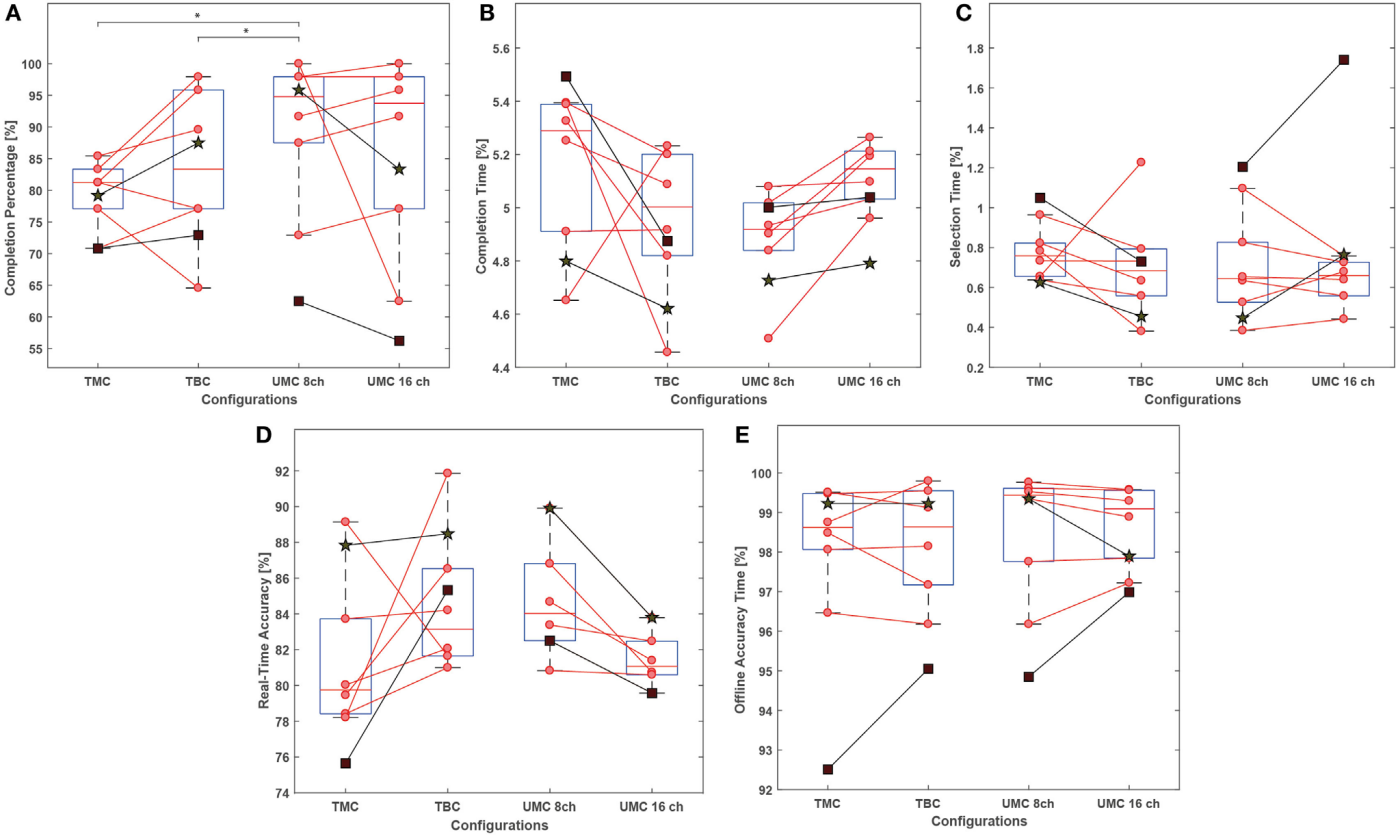
Box plots presenting the results of the comparison of the three electrode configurations in terms of real-time metrics relative [i.e., **(A)** completion percentage, **(B)** completion time, **(C)** selection time, **(D)** real-time accuracy] and **(E)** offline accuracy. The boxplots represent only the data relative to non-amputated subjects. The line in the center of the boxes indicates the location of the median, the upper edge indicates the third quartile, the bottom edge represents the first quartile, and the whiskers indicate the data range. Along with every boxplot, it is possible to the mean value for each subject. Red dots represent non-amputees, while the square and a star marker represent the data points for the transtibial and the transfemoral amputee subject, respectively. Statistical significance (*p* < 0.05) is marked by the *.

**Figure 6:**
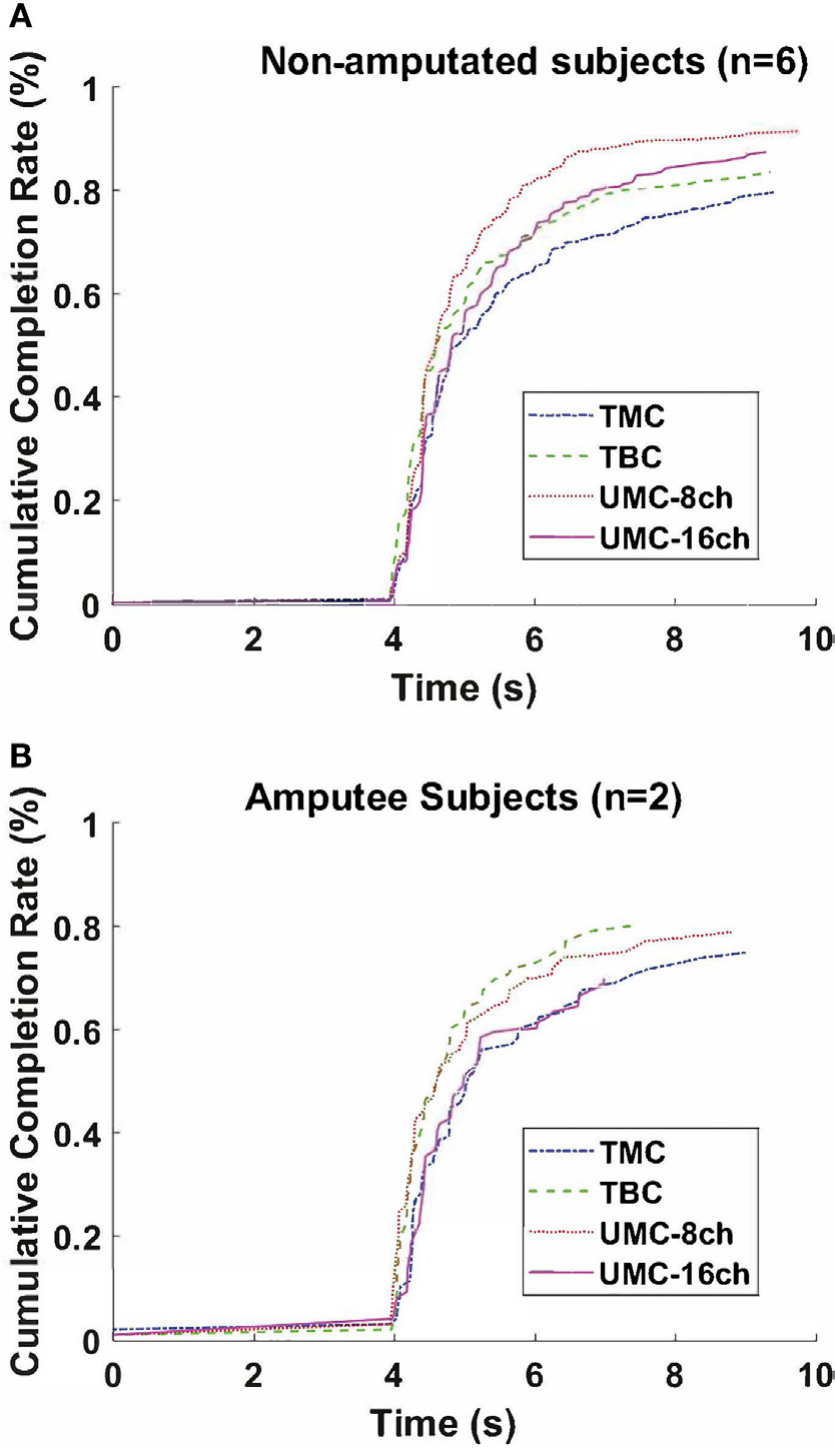
Cumulative completion rate for **(A)** non-amputated and **(B)** amputated subjects.

Table 1 shows the results of the real-time tests as mean values and related SEs. For non-amputees, the real-time performance metrics and the offline accuracy are also presented in boxplots. In addition, data points representing the mean over the motions for amputees and non-amputees are plotted on top of the boxplots, and the pairs of the dependent samples are connected by lines (Figure 5). Finally, Figure 6 shows the cumulative completion rate for both non-amputees and amputees, which represents the percentage of motions completed as a function of time.

**Table 1:** Performance metric mean values (SE) for each configuration: targeted monopolar configuration (TMC), targeted bipolar configuration (TBC), untargeted monopolar configuration with 8 channels (UMC-8 ch), and untargeted monopolar configuration with 16 channels (UMC-16 ch).

Performance metric	TMC		TBC		UMC-8 ch		UMC-16 ch	
	Amputee (*n* = 2)	Healthy (*n* = 6)	Amputee (*n* = 2)	Healthy (*n* = 6)	Amputee (*n* = 2)	Healthy (*n* = 6)	Amputee (*n* = 2)	Healthy (*n* = 6)
Completion rate %	75.0 (4.2)	79.8 (2.1)	80.2 (7.3)	83.7 (5.3)	79.1 (16.6)	91.3 (4.1)	69.8 (13.5)	87.5 (6.0)
Real-time accuracy %	81.7 (6.1)	81.5 (3.0)	86.9 (1.6)	84.6 (2.9)	86.0 (1.6)	84.7 (2.3)	83.9 (2.3)	81.4 (1.1)
Completion times	5.15 (0.35)	5.15 (0.12)	4.75 (0.13)	4.95 (0.12)	4.86 (0.14)	4.88 (0.08)	4.91 (0.12)	5.13 (0.05)
Selection times	0.84 (0.21)	0.77 (0.05)	0.59 (0.14)	0.72 (0.12)	0.83 (0.38)	0.69 (0.10)	1.25 (0.49)	0.88 (0.05)

The statistical testing for the comparison of TMC to TBC did not reveal any significant differences in the metrics for evaluating the performance in real time (completion percentage: *p* = 0.37; selection time: *p* = 0.43; real-time accuracy: *p* = 0.31; completion time: *p* = 0.43) or offline (offline accuracy: *p* = 0.68). Nevertheless, TBC performed better in the majority of the cases when considering the pairs between the two samples (data points connected by lines). A larger sample size could have likely revealed a significant difference.

In comparing UMC (eight channels) to TBC, a significant effect was found for the completion percentage (*p* = 0.002), while the remaining metrics presented no significant differences (selection time: *p* = 1; real-time accuracy: *p* = 0.81; completion time: *p* = 0.58; offline accuracy: *p* = 0.73). Similarly, the comparison between UMC and TMC yielded a significant difference in the completion percentage (*p* = 0.002), but not in the other metrics (selection time: *p* = 0.39; real-time accuracy: *p* = 0.13; completion time: *p* = 0.13; offline accuracy: *p* = 0.48).

Finally, the investigation conducted of UMC revealed that 16 channels did not have any improvement over the performance of the electrode configuration with just 8 channels, and no significant differences were found (completion percentage: *p* = 0.56; selection time: *p* = 0.56; offline accuracy: *p* = 0.68), even though real-time accuracy and completion time were better with 8 channels, as seen from the low *p*-value and the pairwise visual inspection in Figure 6 (real-time accuracy: *p* = 0.03; completion time: *p* = 0.03).

### Part II

**Figure 7:**
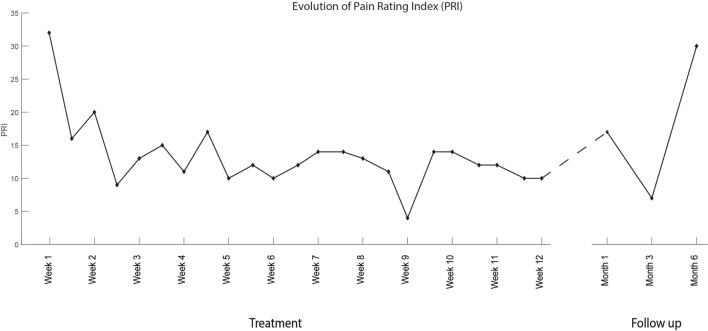
Evolution of the Pain Rating Index (PRI) over the course of the treatment and in the follow-up period (6 months). The PRI is calculated as the sum of the scores (0–5) assigned to the pain descriptors of the Short Form of the McGill Pain Questionnaire (SF-MPQ). The SF-MPQ was administered at the beginning of each treatment session twice per week (left hand side of the dashed line) and at 1, 3, and 6-month follow-up (right hand side of the dashed line).

**Figure 8:**
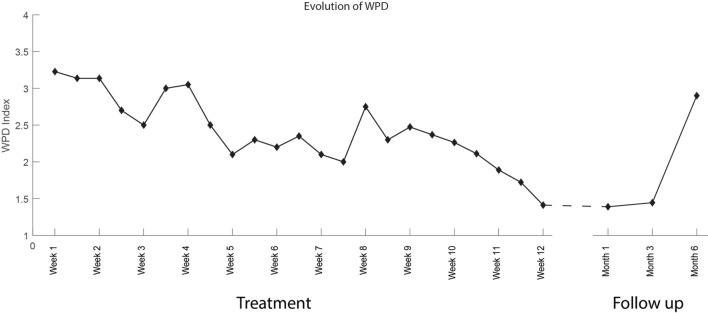
Graph representing the value of the Weighted Pain Distribution (WPD) throughout the 23 treatment sessions and at 1, 3 and 6-month follow-up (right hand side of the dashed line). The WPD is calculated as the sum of the scores (0–5) weighted on the total time spent awake.

**Figure 9:**
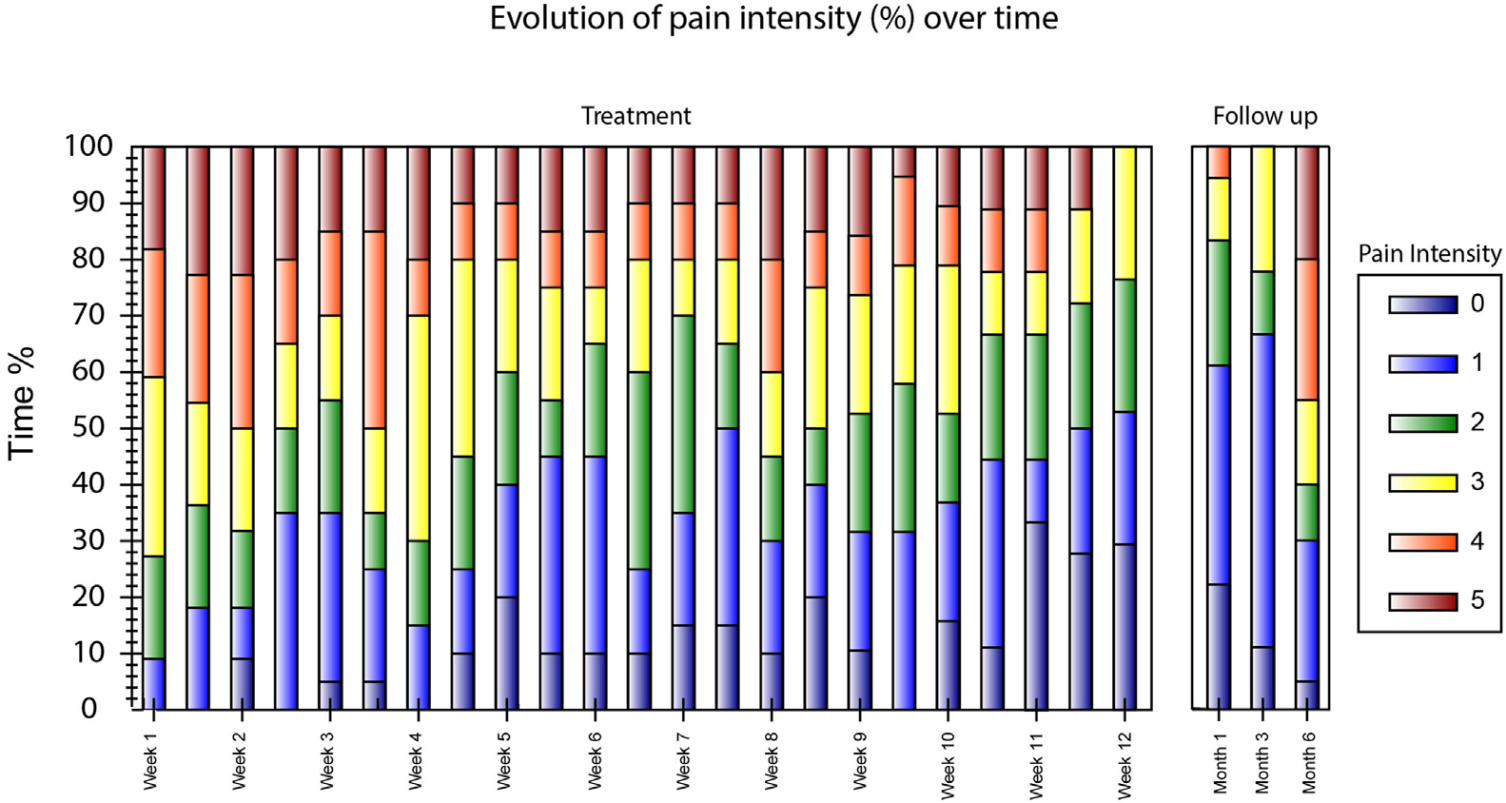
Weighted pain distribution (WPD) bar graph. Each bar represents a treatment session or a follow-up interview. The pain rating is from 0 to 5 where 5 (red) is the worst possible pain.

**Figure 10:**
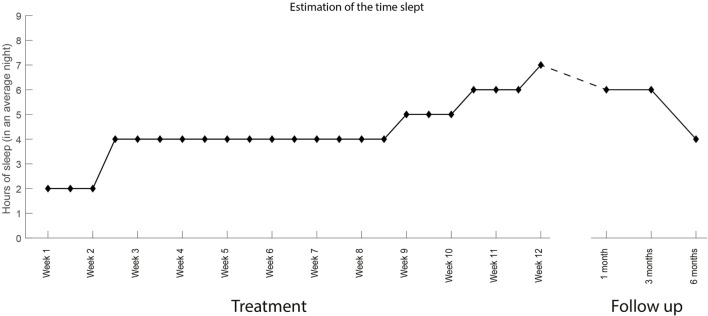
Time slept as estimated by the subject over the course of the treatment and during the follow-up period.

The interventions took place between January 28, 2016 and April 19, 2016. The patient was initially able to control proximal movements (knee flexion/extension, hip rotation medial/lateral) in only 1 degree of freedom. By the end of the treatment, the patient had acquired control over the entire lower limb, including toes, and was able to exercise up to 4 degrees of freedom within the same session (Video S1 in Supplementary Material). Between the first and the last treatment session, an overall reduction of PLP intensity was measured by all metrics. PLP intensity decreased by 2 points on the NRS scale (from 4 to 2, 50%) and by 22 points in PRI (32 to 10, 68%) (Figure 7). A positive change was also reported in the time-varying profile of PLP, in which the WPD decreased by 1.8 points (from 3.2 to 1.4, 57%) by the last treatment session (Figure 8). The progress in pain reduction, presented as distribution of pain over time, is presented in Figure 9, and the estimated time slept is presented in Figure 10. In particular, the higher-intensity PLP (pain levels of 4 and 5), usually present in the evening and at night, reduced considerably over time. This was accompanied by an increase in length and quality of sleep from 2 h per night with interruptions to 7 h without interruptions. The pain location remained constant throughout the entire treatment period (in the foot), and the phantom limb maintained the same dimensions it had at the beginning of the treatment, thus being of the same length as the normal leg. The patient noted an improvement in quality of life since the start of the treatment, with less tiredness, improved mood, and regained ability to drive for long distances (>200 km at a time, which was not possible before). Moreover, both family and patient observed a reduction in the use of the neurostimulator during the day.

From Figures 7–10, it is also possible to see the profile of PLP after the end of the treatment, as recorded at the follow-up interviews 1, 3, and 6 months after. The positive effects of the treatment were retained at the first and second follow-up interviews but had almost vanished by the sixth month.

## Discussion

The aim of this study was twofold. First, we wanted to investigate the performance of two alternative electrode configurations to conventional bipolar targeted recordings in terms of real-time metrics. Second, we evaluated *PME* as a treatment of PLP on lower-limb amputations in a chronic intractable case.

In the first part of this article, we showed that classification is possible similarly in all of the three configurations. Looking at the comparison between TMC and TBC in the boxplots of Figure 5, the latter performed better in most cases. A possible explanation of this result is that the distance between the electrodes, and the CCE in TMC, is generally larger than the inter-electrode distance for TBC. This could result in an increase of crosstalk picked up by the electrodes and CCE, yielding lower SNR, as our previous study showed (23). Conversely, the distance between the electrodes and the CCE in the UMC was reduced, possibly rendering fewer disturbances in the signals, thereby explaining the better performance.

It is worth noticing that the UMC with 16 channels did not outperform the same configuration with just 8 channels. On the contrary, it might appear that, when considering real-time accuracy and completion time, fewer channels improved the performance.

Besides real-time performance of the classifier, there are secondary factors that can be taken into account to determine which electrode placement method should be preferred for a clinical application. First, TBC might not be an option when dealing with patients with short stumps, as not all the muscles required for targeted configurations might be available. Second, the targeted electrode placement can be difficult and time consuming because of the difficulty of identifying the correct muscles, due to excessive soft tissue, weakness, or muscle relocation, even when the muscles are available. Third, the use of bipolar electrodes requires parallel alignment to the muscle fibers for optimal recordings (33), as well as avoiding innervation zones (34). Parallel alignment in differential measurements is recommended because this is the direction of the propagation of the action potential. However, this alignment is difficult to achieve in muscle fibers forming a pennation angle (such as the quadriceps). Altogether, sEMG signal acquisition in the lower limbs could be facilitated by placing the electrodes in monopolar configurations (UMC and TMC). This configuration is insensitive to the fiber orientation and position of the electrode, with respect to the innervation zone. Moreover, we show that it is not necessary to target all the superficial muscles of the thigh, even when available. UMC yielded real-time classification accuracy comparable to the targeted configurations (TMC and TBC). However, optimizing the targeted electrode placement by identifying the active areas of the stump muscles can improve the quality of the MPR in amputee subjects.

Altogether, UMC or TMC, with CCE made of conductive fabric, was beneficial for implementing a rehabilitation system. In addition to faster and easier electrode placement, such configurations also need only half the pre-gelled adhesive electrodes normally used in a bipolar configuration. This means an economic advantage, in addition to reducing material waste.

Moreover, the use of the CCE of conductive fabric opens possibilities for developing solutions made entirely of wearable smart textiles, which would allow patients to easily take them on and off. In addition, a textile solution could be reused and easily be adapted for different anatomies without changes in the design (35).

The second part of the paper was dedicated to evaluating *PME* as a strategy to treat PLP in a subject with lower-limb amputation. In accordance with previous studies on upper limbs (4–6), improvement was found in all the metrics used for pain evaluation following treatment by *PME*. Conversely, PLP was not eliminated completely, despite the fact that the intervention took place over a longer period of time and follow-up interviews revealed that the positive effects almost vanished within 6 months, as opposed to what was demonstrated in the previous clinical trial. Overall, this might indicate that more sessions are required in case of PLP in the lower extremities, or that the contribution of augmented reality could induce more rapid, longer-lasting changes.

Nevertheless, we showed that the realistic visual feedback induced by augmented reality was not essential to obtain pain reduction *via PME* treatment, raising doubts as to whether or not, a more realistic visual illusion concerning the virtual limb is necessary to mediate the perception of PLP. Our work and others suggest a relationship between the ability to control movements of the phantom limb and PLP, and therefore we cannot exclude that pain relief could be achieved just by training phantom mobility without appropriate visual feedback. Our previous studies, together with the current one, are limited in this sense due to the lack of an appropriate control group, and additional investigations aimed at unveiling these aspects are required.

Although not quantified, we observed morphological changes in the stump related to regained muscular mass. These changes were accompanied by improvement in voluntary control of the phantom limb, also not recorded by any direct measure, but clearly indicated by the ability to control an increasing number of degrees of freedom of the virtual limb. It is possible that structural alteration of the stump was accompanied by functional and neurophysiological variations, accounting for the effects that we observed on PLP. In the future, studies should quantify morphological changes in the stump, improvements in phantom motor control, alteration of sensorimotor cortical maps, and how these relate to PLP.

Finally, the use of a CCE for monopolar recording may allow for faster electrode placement, which means that more time can be spent in the treatment rather than in the setup. Moreover, using the monopolar configuration also implies that roughly 200 Ag/AgCl pre-gelled electrodes were spared in this particular case study.

## Conclusion

In the first part of this work, we demonstrate the possibility to use different techniques to acquire sEMG signals suitable for successful MPR of lower-limb movements in non-weight-bearing conditions. We concluded that monopolar recordings, enabled by a single differential electrode around the leg, seem a viable solution for a rehabilitative application. Future work will focus on further development of the system to make it more user-friendly.

In the second part, we investigated the efficacy PME in reducing chronic, intractable PLP on a subject with lower-limb amputation. The results were limited to one subject but were positive and put forward the need to investigate in a wider population to determine if PME, facilitated by MPR and VR, can effectively reduce PLP in the lower limb.

In conclusion, the results of this research give us grounds to continue the work on our long-term goal of implementing a system for treating PLP based on *PME* for subjects with both upper- and lower-limb amputations.

## Ethics Statement

This study was carried out in accordance with the recommendations of the *Handbook for Good Clinical Research Practice*, by the World Health Organization. All subjects gave written informed consent in accordance with the Declaration of Helsinki. The regional ethical committee of Västra Götalandsregionen approved this study.

## Author Contributions

EL and MO-C designed the studies and the electrode configurations. EL performed the literature review, conducted the study on the electrode configurations, performed the interventions for the *phantom motor execution* treatment, analyzed the results, and drafted the manuscript. MO-C developed the motion prediction technology (software). EM designed and developed the hardware. MO-C and BH supervised this research and revised the manuscript. All the authors have read and approved the final manuscript.

## Conflict of Interest Statement

MO-C was partially funded by Integrum AB, a for-profit organization that might commercialize an improved version of the technology here described. The core technology used in this study has been made freely available as open source by MO-C and EM (machine learning, virtual reality, and electronics). EL, EM, and BH declare no competing interests.

## Supplementary Material

Video S1 The subject performing the target achievement control (TAC) test with 4° of freedom during the phantom motor execution (PME) treatment.
